# Perspectives of Non-Pharmacy Professionals in Long-Term Care Facilities on Pharmacist-Involved Medication Management in South Korea: A Qualitative Study

**DOI:** 10.3390/ijerph16111977

**Published:** 2019-06-04

**Authors:** Arim Kwak, Euni Lee, Jung Mi Oh, Eunhee Ji, Kyungim Kim

**Affiliations:** 1 College of Pharmacy, Korea University, 251l Sejong-ro, Sejong 30019, Korea; arimkwak@gmail.com; 2 Seoul National University, 1 Gwanak-ro, Gwanak-gu, Seoul 08826, Korea; eunilee@snu.ac.kr (E.L.); jmoh@snu.ac.kr (J.M.O.); 3 College of Pharmacy and Research Institute of Pharmaceutical Sciences, College of Pharmacy, Gachon University, 191 Hambakmoero, Yeonsu-gu, Incheon 21936, Korea; ehji@gachon.ac.kr

**Keywords:** pharmaceutical care, medication management, long-term care facility, public health, geriatric, South Korea, qualitative study

## Abstract

Despite a rapid increase in both the number of long-term care facilities (LTCFs) and their residents in recent years, the concept of pharmacist-involved medication management is relatively new in South Korea. The objective of this study was to identify the perspectives of non-pharmacy professionals regarding the development of pharmacist-involved medication management in LTCFs. Employing a snowball sampling strategy, this study relied on semi-structured, one-on-one, in-depth interviews with twelve non-pharmacy professionals in LTCFs. The inductive thematic analysis and the constant comparative method were employed for the analysis. Participants revealed the need for pharmacist-involved medication management systems in LTCFs at the intrinsic and environmental levels. Through pharmacist-involved medication management, participants desired “medication review/reconciliation” and “pharmaceutical education/counseling”. The barriers to be overcome included “the authorization of pharmacists’ roles”, “the financial stability of LTCFs”, “role awareness among coworkers”, and “the professional development of pharmacists”. In this study, we advanced our understanding of non-pharmacy professionals’ perceptions of pharmacist-involved medication management in LTCFs. The results of this study can be applied in other Asian countries where the development of pharmacist-involved medication management for the institutionalized elderly is relatively new.

## 1. Introduction

Residents of long-term care facilities (LTCFs) are known to have multiple clinical conditions requiring drug therapy. Because of their susceptibility to drug-related problems caused by physiologic changes with the aging process and the high prevalence of multiple medications (polypharmacy), the need and the importance of comprehensive medication management services (e.g., prescription, medication review, dispensing, storage) have been described [1,2]. The role of pharmacist-led or pharmacist-involved medication management (PIMM) serving this vulnerable population has been documented in most developed countries [3,4,5], and other studies have also shown that these services improved the overall quality of medical care and reduced potentially inappropriate medication use and drug costs in LTCFs [6,7,8,9]. The U.S. implemented extensive legislation in 1990, which mandates the monthly reviews of drug regimens for nursing home residents and requires the consulting pharmacist to report any irregularities to the attending physician and the director of nursing [4,5].

In this paper, we address the situation related to the healthcare environment surrounding pharmacy practices in South Korea (hereafter, Korea), one of the fastest aging countries in the world. Seniors aged 65 or older represented 13.8% of the population in 2017 and are projected to represent 41.0% by 2060 [10]. While the demand for LTCFs has grown over the last decade as a consequence of the accelerated aging of Korean society, pharmacists carrying out medication management in LTCFs or the infrastructure for the service are rarely available at present. LTCFs in Korea can be categorized in two ways—“geriatric care hospitals” (GCHs) and “assisted living facilities” (ALFs). In an ALF where nurses or social workers mainly provide assistance with the activities of daily living, residents are usually provided healthcare services from a contracted physician who visits a facility biweekly or from other outside physicians. As there are no regulatory requirements regarding pharmacists working in ALFs, medications are often delivered by multiple providers without any meaningful care coordination. A GCH is similar to a “skilled nursing facility” in some western countries including the U.S., where not only assistance with the activities of daily living but also medical care services are provided. Unlike an ALF, a GCH has attending physicians and registered nurses on staff 24 h a day. The current regulation in Korea allows a GCH with less than 200 beds to employ one pharmacist who works only 16 h per week and nurses or physicians to fill prescriptions while the pharmacist is off duty [11]. With the current regulatory climate allowing a short time frame of 16-h weekly service for pharmacists, the majority, if not all, of professional activities of the pharmacists would be spent on filling prescriptions without sparing sufficient time to provide cognitive services such as medication review and therapy recommendations. 

Given that the concept of PIMM in LTCFs is relatively new in Korea, it is important to understand perceptions of non-pharmacy professionals about PIMM services. The aim of this study was to gather thoughts and opinions from non-pharmacy professionals to inform the development of PIMM in LTCFs in Korea.

## 2. Materials and Methods 

This study adopted the methodology of “Consolidated Criteria for Reporting Qualitative Research” (COREQ-32 checklist) [12] (see Table A1).

### 2.1. Study Design and Participants

Semi-structured, one-on-one, in-depth interviews were conducted for this study. The participants included a range of physicians, nurses, and social workers currently working at LTCFs in Korea to ensure a broad understanding of medication management from those directly involved in geriatric care in LTCFs. Potential interviewees were recruited by a snowball sampling method and asked to participate in the study via telephone, email, or visit. Participants were eligible if they had a minimum of one year of clinical experience in LTCFs, were licensed in their respective fields, and agreed to discuss their personal views regarding PIMM in LTCFs. The recruitment and interviews were conducted simultaneously, and the recruitment ended when saturation had been reached in terms of the views expressed, with similar opinions and concepts repeatedly recurring in the study topic. This study was approved by the Human Research Ethics Committee of the Ministry of Health and Welfare, Korea (IRB No. P01-201406-SB-03). Interviewees gave informed written consent before their participation. 

### 2.2. Researchers and Interviewer

A faculty member in pharmacy (the principal investigator, K.K.) who has conducted research in pharmacy practice led the interviews. The interviewer was trained in conducting interviews. No prior personal relationships between the interviewer and the participants existed.

### 2.3. Data Collection

The interviews were conducted between August and October 2014. An interview guide was pre-developed and reviewed by specialists from the related field. The guide included interview questions regarding perspectives on the current medication management system in LTCFs and the development of PIMM (Table 1). Pilot interviews were performed with one social worker and one pharmacy graduate student, but the interview data were not included in data analysis. Each participant took part in an individual interview in a private room at their workplace and received an incentive after the interview. Before each interview, the definition of “pharmacist-involved medication management” (i.e., a pharmacist’s provision of clinical activities in making patient-centered recommendations on medication management in collaboration with other professionals working in LTCFs) was explained to prevent any confusion around the concept. Participants were also informed that their names and workplaces would be omitted during the transcription of the recordings to ensure confidentiality.

### 2.4. Data Analysis and Reports

All interviews were audio recorded and transcribed verbatim by research staff members. To ensure quality, the interviewer checked for the accuracy of the interview transcripts. A preliminary codebook was developed with its definitions as recommended by DeCuir-Gunby et al. [13]. To this end, the two main researchers (K.K. and A.K.) carefully read all transcripts line by line to inductively identify emergent codes and themes. The original statements of the participants were paraphrased to shorter phrases, and then were reduced to short and meaningful keywords. Afterwards, superior codes and themes were determined and summarized. The codebook was refined with repeated transcript coding by consultative discussions with the other authors. For the analysis, transcripts were reviewed again and double-coded by the two main researchers. Results were structured using thematic domains, codes, and compelling quotes to draw conclusions. In addition, using the constant comparative method, prior interviews coded were constantly re-analyzed in light of codes that emerged in later analyses. Throughout the process, any disagreement was resolved either by discussion between the two main researchers, or by considering the opinion of additional researchers (E.J., E.L., and J.M.O.) to reach consent. For reporting, the original language was translated into English by two independent bilingual translators. For validation purposes, the data were double-checked by back-translating English to Korean. The reference at the end of each quotation indicates the participant number followed by the paragraph numbers where the quotation occurs in the transcript, for example, “Participant #3, 109–111”.

## 3. Results

A total of 12 non-pharmacy professionals working in nine LTCFs participated in this study. A summary of the participants’ characteristics is shown in Table 2. Participants’ ages ranged between 34 and 64 years, with a mean of 51.2 years. The average length of work experience of the participants in LTCFs was 5.9 years (range, 2.0–9.0 years). In-depth interviews lasted between 41 and 88 min per participant, with a mean of 57 min.

Three main themes (Needs, Expectations, and Barriers) and eight codes emerged from the analysis of the participants’ interviews (Table 3).

### 3.1. Needs

For the theme “the need for pharmacist-involved medication management”, participants in the study appeared to have positive responses. Participants revealed their thoughts in two ways: (1) intrinsic factors and (2) environmental factors. The intrinsic factors revolved around characteristics of geriatric residents (e.g., necessity of medication use, polypharmacy, and diverse responses to medications). The environmental factors included routines, policies, and operating systems in LTCFs (e.g., physicians as sole decision makers, the limited role of pharmacists, the lack of awareness of practitioners, and the heavy workload of the staff).

#### 3.1.1. Intrinsic Factors

##### Necessity 

All participants expressed their belief that medication management is a crucial issue for geriatric care in LTCFs since medications are essential for almost all residents to maintain their health status. One participant’s comments below illustrate how they perceive the importance of medication in LTCFs.

*(Medication is) absolutely important. Whether the elderly people die or live depends on the medications.* (Participant #1, 269, 270)

##### Polypharmacy

Almost all participants stated that residents living in their LTCFs took a lot of medications concurrently (polypharmacy) to manage their multiple comorbidities. In the example below, one participant expressed concern about unnecessary medication use, potential adverse drug reactions, and drug–drug interactions.

*We were shocked (by the number of the medications). Some residents brought so many drugs with themselves when they were discharged from their hospitals, about 15 medications or even more than 20 drugs at a time. In my view, that’s pretty excessive. That’s too many. Some residents brought 3 or 4 types of expectorants even though they didn’t have cough or sputum.* (Participant #2, 119–121)

##### Diversity

A couple of participants described an inter-individual or intra-individual variety of responses toward medications and were concerned with the difficulty of predicting the efficacy and safety of the medications administered.

*Even when we use a simple drug, like acetaminophen, some people respond to only 300 mg while others do not respond to even 2 g a day. […] In my view, older people may show various responses to the same drug, dramatic effects or none, even if the dose is based on the textbook.* (Participant #3, 109–111, 114–116)

#### 3.1.2. Environmental Factors

##### Physicians as Sole Decision Makers

All participants agreed that they placed primary responsibilities on the physicians regarding medication use in LTCFs. A number of participants mentioned that the care team was entirely dependent on the physicians’ decisions and was “almost pushed around, in a way everything is done by the doctors” (Participant #4, 224–226). In this regard, most participants were doubtful about the physicians’ competence in medication in geriatric care.

*Realistically speaking, (among physicians) they don’t really know much about drugs on the market, and also if it’s not within their specialties, they don’t really know the details about the drug or interactions of the drugs.* (Participant #3, 161–163)

##### Limited Role of Pharmacists

Regardless of their professions and working environments, almost all participants stated that they rarely had an interaction with a pharmacist in person or even by telephone or email to discuss medication-related issues for residents. A couple of participants explained that the main reason to contact pharmacists was to deal with administration issues such as storing controlled substances. Participants repeatedly mentioned that the LTCF pharmacists’ role for their residents was limited to dispensing medications at the facility. One participant working in a GCH emphasized the short working hours of pharmacists and the hardship associated without full-time pharmacists.

*Only 16 hours. Because pharmacists work for a very short period of time, pharmacists are not involved in drug management at all […] Someone should take the role of being a bridge between doctors and residents, or other staff, but it’s quite burdensome for us to take that responsibility because we are not experts in medications […] In my view, pharmacists are too busy filling prescriptions during their shifts.* (Participant #5, 51, 52, 63, 64, 101)

##### Practitioners’ Lack of Awareness

Participants expressed a deep concern about “the zero interest in or knowledge of drugs” (Participant #3, 328) of the practitioners such as nursing assistants and caregivers who provide assistance with the administration of medicine to residents. They worried that this could lead to inadequate medication administration or illegal “regimen change by imprudent practitioners who lack the proper knowledge of medication” (Participant #6, 60, 61) without a prescriber’s permission.

*(Caregivers) sometimes are careless about medications. “Skipping once won’t hurt” or when they drop a pill while opening the drug packet, they show careless behavior thinking, “it’s just one pill, it’s okay” by throwing the pill in the trash can.* (Participant #1, 316–318)

### 3.2. Expectations

Participants expressed positive attitudes toward PIMM in LTCFs. The main services that participants expected from PIMM included “medication review/reconciliation” and “pharmaceutical education/counseling”.

#### 3.2.1. Medication Review/Reconciliation

In Korea, it is possible for LTCF residents to receive prescriptions from multiple physicians inside and outside the facilities in order to meet personal therapy needs. Under this patchwork of prescriptions, participants agreed that “a proper medication verification process, reviewing drug–drug interactions, and monitoring if the prescriptions are filled correctly” needs to be implemented (Participant #7, 231–233). In addition, they revealed the need for medication reconciliation that refers to a resident’s complete medication regimen review at the time of admission, transfer, and discharge and comparing it with the regimen being considered for the new setting [14]. Key issues such as “duplicated therapy” and “drug–drug interactions” were frequently reported.

*Just doing a lot of drug–drug interaction reviews can be adequately helpful.* (Participant #8, 330)

*The pharmacist’s drug management could include comparing the resident’s condition and the drugs currently being prescribed along with making sure that no unnecessary drugs are being used on a resident, (and) duplicated therapy.* (Participant #1, 245–247)

#### 3.2.2. Education/Counseling

Participants also acknowledged the expectations from pharmacists’ education/counseling and emphasized the word “repetition”, stressing the importance of continuous implementation. The need for the education of staff such as physicians, nurses, social workers, caregivers, and nursing assistants included “the importance of medication management and compliance”, “information about commonly used drugs”, and “drug administration instructions”.

*Pharmacists need to question if the healthcare team members know the importance of the medication and to provide education courses twice a month or at least every quarter for re-emphasizing the importance of the drug and its management over and over again.* (Participant #1, 453–456)

Additionally, participants suggested the need for pharmacists to make a prioritized visit “maybe not every resident, but just 1–2 priority residents” (Participant #5, 280, 281) and to provide counseling to the residents about “drug administration” and “adverse drug reactions”.

*If pharmacists can explain adverse drug reactions, so if it can lead residents to recognize and notice (adverse drug reactions) early, it will be good for doctors to treat it. “How to take medicines appropriately” and “adverse drug reactions”, I think that these two points are really, really important.* (Participant #9, 179–182)

### 3.3. Barriers

Participants described a wide range of barriers to be overcome for the development of PIMM in LTCFs: the authorization of pharmacist roles, the financial stability of LTCFs, the professional development of pharmacists, and role awareness between coworkers.

#### 3.3.1. Authorization of Pharmacist Roles

The majority of participants mentioned that they felt the absence of pharmacists in medication management for LTCF residents, but they expressed mixed views about the authorization of pharmacists’ role in LTCFs. Some participants emphasized that it was necessary to establish more “official duties” for LTCFs or pharmacists, which included an increase in the working hours of pharmacists, the number of pharmacists needed, or designating the roles of pharmacists in LTCFs.

*Whether we want to do it or not, it is necessary to establish pharmacists’ roles (in LTCFs) by law and then we can abide by the rules. In fact, it is a desirable direction for promoting public health.* (Participant #3, 368–370)

In contrast, a couple of participants revealed hesitation about the authorization of pharmacists’ roles in LTCFs by regulatory means. The participants worried that official regulations that do not reflect the current circumstances could make it harder for them to manage the facilities.

*Practically speaking, it is difficult. We already receive a regular evaluation every two years, there are close to 100 evaluation items, it’s really hard for us. […] If there is something new in the regulations without any consistent direction, it can be very difficult.* (Participant #11, 388–390, 393–394)

#### 3.3.2. Financial Stability of LTCFs

All participants discussed the financial difficulty of LTCFs in employing pharmacists to provide more clinical activities under the current system where most LTCFs are run privately. Some participants pointed to the lack of policy or remuneration system to support PIMM in LTCFs as a substantial barrier. They were concerned that without additional funding, neither LTCFs nor pharmacists would be willing to increase their economic burden or tasks, respectively, as it is financially challenging for LTCFs to pay pharmacists for the provision of pharmaceutical care services.

*We are somewhat dependent on money. It is, in fact, quite a burden for facilities to hire a pharmacist by spending a lot of money [...] The government should not be talking about developing pharmacists’ roles but providing actual support for the employment of pharmacists. It seems like the burden ultimately is on facilities because the government is simply laying the blame on facilities without providing any support. In some way, it is necessary to have a support system at the government level.* (Participant #5, 104, 105, 112–116)

#### 3.3.3. Professional Development of Pharmacists 

Participants highlighted the qualifications of pharmacists related to geriatric care in the current context. Some participants worried that the pharmacists currently working in LTCFs would not have enough competence “to verify and closely examine commonly prescribed medications and drug interactions” (Participant #7, 409). They emphasized the importance of “specified knowledge and experiences” because medication management in geriatrics requires not only a fundamental understanding of age-related characteristics but also various clinical experiences in related fields. In addition, the importance of active mindsets “about how pharmacists develop knowledge and become interested in it” (Participant #3, 381, 382) was addressed.

*What is important will be the work experience. A pharmacist should be experienced because geriatric medication differs according to each resident. It is important not to be theoretical but also to have clinical experience because it is important to understand the disease conditions, characteristics, and progression in each resident. In that respect, I think that some practical experiences as well as theoretical education are needed.* (Participant #6, 243–247)

#### 3.3.4. Role Awareness among Coworkers 

Participants touched upon the importance of “mutual understanding” between coworkers in LTCFs. They admitted that the role of pharmacists often overlapped with the role of other healthcare professionals in LTCFs, but there clearly is a specific domain of practice for pharmacists. They believed that there is a need to understand each other to develop an ideal PIMM in LTCFs.

*It would be necessary to recognize, value, and share each profession’s role in order to specify what we have talked about so far.* (Participant #4, 551, 552)

## 4. Discussion

This study investigated how non-pharmacy professionals working in LTCFs perceive the development of PIMM in Korean LTCFs. In this study, it is evident that there is a need for PIMM, and “medication review/reconciliation” and “pharmaceutical education/counseling”. For the development of effective PIMM, participants discussed some barriers to be overcome at the government, facility, and pharmacist levels.

Throughout the interviews, we verified the need for PIMM in LTCFs as identified by non-pharmacy professionals. Participants’ commentary showed how they specifically recognized the importance of medication management for their LTCF residents (intrinsic factors) and perceived the limitations of the current medication management system in LTCFs (environmental factors). This finding had to be noted because the reported prevalence of potentially inappropriate medications was high among LTCFs in Korea. The studies found that the incidence of at least one potentially inappropriate medication was 41.4–58.2% in elderly individuals who resided in LTCFs [15,16]. Indeed, demands for the improved integration of pharmacists for better medication management in LTCFs have emerged in recent years in Korea. 

Participants mentioned “medication review/reconciliation” and “pharmaceutical education/counseling” as necessary components of PIMM in LTCFs. Pharmacists’ contributions in medication reviews and education about medication management are the most important parts of their clinical activities, and it is well known that those activities of pharmacists have improved the safety of prescribing medications in LTCFs [17,18,19]. The expectations mentioned through these interviews, however, are relatively simple when compared to those in a previous study that investigated the perception and need for pharmaceutical care services among non-pharmacy professionals working in teaching hospitals [20]. Given that pharmacists have much more active clinical roles in teaching hospitals than in LTCFs, the low level of expectations from PIMM in the current study may be due to no experiences in interacting with pharmacists and less awareness of pharmacists’ potential roles in LTCFs. Tan et al. supported this concern with the results of their study in primary-care settings; increasing the physical proximity between non-pharmacy professionals and pharmacists is cited as a major element in enhancing interprofessional collaborations [21]. McDonough and Doucette’s collaborative working relationships model could be one of the ways to resolve the aforementioned issue [22]. This model is composed of communication opportunities, sufficient time to take a step-wise approach, and the establishment of clearly defined roles and shared responsibilities.

This study provides a deeper understanding of some of the practical issues that must be addressed when developing PIMM in LTCFs. First of all, the study pointed to how the government’s action was needed regarding the authorization of the pharmacist’s role in LTCFs, the qualification of a geriatric-specialized pharmacist, and the payment/remuneration system. In Korea, where LTCF residents often experience potentially inappropriate medication, establishing the role of qualified pharmacists as pharmaceutical care service providers is vital. In this vein, the government needs to refer to other countries where the role of pharmacists as service providers of pharmaceutical care in LTCFs and an appropriate remuneration system are regulated in the long-term care insurance system such as in Japan (i.e., long-term care insurance) and Germany (i.e., care insurance). 

Second, the participants of this study brought up the need for “the professional development of pharmacists” in which “specified knowledge and experiences” and “active mindset” are included. Since geriatric care is complicated by the significantly large number of medications that patients take to manage various chronic and acute conditions, pharmacists working in LTCFs are expected to have not only specialized geriatric knowledge but also extensive work experience. Geriatric content in the current education of pharmacy schools, however, is still disproportionate to the continuing increase of the elderly population in Korea [23]. Developing geriatric competencies across the continuum of pharmacy education in college and ensuring geriatric-specialized training to be provided for geriatric care after pharmacy school are needed to be considered. 

**Figure 1:**
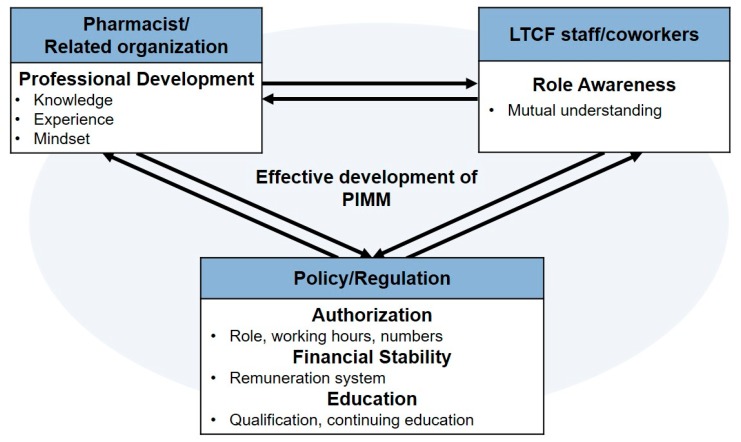
Essential elements and their interactions for the effective development of pharmacist-involved medication management (PIMM).

Third, for “role awareness among coworkers”, previous studies in the U.S. and Canada have reported that the lack of role awareness, poor communication, and insufficient collaboration are associated with a decreased acceptance of pharmacists’ roles within primary healthcare teams [24,25]. Interprofessional education where two or more healthcare professionals are learning together could be one of the ways to resolve the aforementioned problem. In an interprofessional education environment, students are prepared to collaborate more effectively with other team members by understanding and valuing each professional’s roles and responsibilities and focusing on achieving one’s own competency in collaborative work. The issues mentioned above can be overcome not only by the individual efforts of the government, pharmacist, or LTCF, but also by the simultaneous efforts of all stakeholders (Figure 1). 

The strengths of our study should be mentioned. First, the interviewer had no prior personal relationships with the participants. The participants were able to objectively provide various accounts of the current status of PIMM at its budding stage and provided an opportunity to develop future directions of the services in such an environment. Second, the results from this study are not confined to the LTCF setting in Korea, but may be applicable to those in other Asian countries as well. Although most Asian countries are rapidly aging [26], the development of comprehensive LTCF services and development of PIMM for geriatrics are relatively new, with the exception of some advanced countries [27].

The findings of this study should be interpreted with caution due to some limitations. First, the internal validity might suffer from the fact that the interviewer was from the faculty of a college of pharmacy, which could have prevented informants from sharing divergent opinions that criticized pharmacists. For this, it is believed that using open-ended questions and allowing the informants to share real-life experiences contributed to the validity of results for the sample and the phenomenon being studied. The interviewer also tried to encourage the informants to speak freely during the interviews. Second, the study sample included a relatively small number of participants including non-pharmacy professionals. Nonetheless, we believe that such limitations were minimized by the recruitment of participants from diverse backgrounds until data saturation was achieved.

## 5. Conclusions

This study has elucidated an understanding of the perceptions of non-pharmacy healthcare professionals regarding PIMM in LTCFs in Korea. Appropriate policy and regulations, professional development of pharmacists, and role awareness between coworkers are key elements for the effective development of PIMM in LTCFs in Korea. Future studies investigating the pharmacists’ perceptions would provide more comprehensive views of the successful implementation and methods of improvement for PIMM in LTCFs.

## Figures and Tables

**Table 1:** Interview guide.

**Introduction**
I want to thank you for taking the time to meet with me today. My name is Kyungim Kim. I’m a faculty member at a college of pharmacy and also a pharmacist. I would like to talk to you about your experience in long-term care facilities. The purpose of this in-depth interview is to hear your thoughts and opinions about pharmacist-involved medication management in long-term care facilities. The interview will take less than an hour. This interview will be audio recorded because I do not want to miss any of your comments. Although I will be taking some notes during the session, I can’t write fast enough to get it all down. We are on tape, so please be sure to speak up so that we don’t miss your comments. All responses will be kept confidential. This means that your interview responses will only be shared with research team members and we will ensure that any information we include in our report does not identify you as the respondent. There are no right or wrong answers to my questions. Please feel free to share your opinions. Remember, you don’t have to talk if you do not want to and you may end the interview at any time. Are there any questions about what I have just explained? Are you willing to participate in this interview?
**Questions**
What do you think about the current medication management system in long-term care facilities? How do you collaborate with pharmacists working in long-term care facilities? What do you think about a pharmacist-involved medication management system to improve the quality of medication use in long-term care facilities? - [If positive] What types of action/services do you expect from the pharmacists, if provided? In what way(s) can pharmacists contribute? - [If negative] What factors do you think would make it difficult to implement pharmacist-involved medication management? What requirements do you think would be necessary for the development of pharmacist-involved medication management in long-term care facilities?
**Closing**
Is there anything more you would like to add? Thank you for your time.

**Table 2:** Background information of participants.

Variable	*N*
Gender	
Male	5
Female	7
Age	
30–39	2
40–49	2
50–59	6
60–69	2
Occupation	
Physician	4
Registered nurse	3
Social worker	5
Institution ^a^	
Geriatric care hospital	5
Assisted living facility	8
Practice location ^b^	
Seoul	4
Gyeonggi	8
Years in LTCF practice	
<5	4
5–10	8
Setting	
With on-site pharmacist (part-time)	5
Without on-site pharmacist	7

^a^ One physician was working in both settings. ^b^ Seoul (capital city of South Korea); Gyeonggi (province near Seoul).

**Table 3:** Thematic categories and code definitions.

Thematic Category	Definition
Theme 1	
1. Needs	Situation where a pharmacist-involved medication management is needed
Code	
1.A. Intrinsic factors	Factors that contribute to the need for pharmacist-involved medication management due to the characteristics of the geriatric residents
1.B. Environmental factors	Factors that contribute to the need for pharmacist-involved medication management due to the environment of LTCFs
Theme 2	
2. Expectations	Services expected from the pharmacist involved in medication management
Code	
2.A. Medication review/reconciliation	Medication review during residence or at the time of admission, transfer, and discharge for the provision of appropriate feedback
2.B. Education/counseling	Formal or informal education/counseling about medications for staff members and residents
Theme 3	
3. Barriers	Barriers to be overcome to make pharmacist-involved medication management feasible in practice
Code	
3.A. Authorization	Authorized role or responsibility of pharmacists in LTCFs in developing pharmacist-involved medication management
3.B. Finance	Financial stability of LTCFs in developing pharmacist-involved medication management
3.C. Professional development	Pharmacists’ professional development in providing pharmacist-involved medication management
3.D. Awareness	Mutual understanding/awareness among the staff members in developing pharmacist-involved medication management

## Appendix A

**Table A1:** Description of Study Methodology Using Consolidated Criteria for Reporting Qualitative Studies (Based on COREQ-32 Checklist).

Item	Description
Domain 1: Research team and reflexivity	
Personal characteristics	
1. Interviewer/facilitator	K.K.
2. Credentials	Pharmacist, Ph.D.
3. Occupation	Faculty (College of Pharmacy)
4. Gender	Female
5. Experience and training	Currently conducting research in pharmacy practice in a college of pharmacy; previously worked in a hospital pharmacy and community pharmacy as a pharmacist
Relationship with participants	
6. Relationship established	No prior relationship existed between the interviewer and the participants. Participants were recruited by snowball sampling.
7. Participant knowledge of the interviewer	The participants did not know the interviewer prior to their interview. The interviewer introduced herself and explained the goals of the research prior to their in-depth interview.
8. Interviewer characteristics	The interviewer is a professor at a college of pharmacy and conducts research about the role of pharmacists in geriatric care.
Domain 2: Study design	
Theoretical framework	
9. Methodological orientation and theory	Inductive thematic analysis using a codebook
Participant selection	
10. Sampling	Participants were recruited by snowball sampling.
11. Method of approach	Telephone and e-mail
12. Sample size	Physicians: 4; registered nurses: 3; social workers: 5
13. Nonparticipation	No participant withdrew from participating in the study.
Setting	
14. Setting of data collection	Workplace of participant
15. Presence of nonparticipants	No nonparticipants
16. Description of sample	Presented in Table 3
Data collection	
17. Interview guide	Presented in Table 2. Semi-structured interview guide was written by K.K. and reviewed by J.M.O.
18. Repeat interviews	No
19. Audio/Visual recording	All in-depth interviews were audio recorded and transcribed verbatim.
20. Field notes	Field notes were made during the interviews by A.K. when necessary.
21. Duration	In-depth interviews took 41–88 min per participant.
22. Data saturation	Data were collected until thematic saturation was reached and no new themes were emerging.
23. Transcripts returned	The transcripts were returned to each participant, and the participants were asked to check them for accuracy.
Domain 3: Analysis and findings	
Data analysis	
24. Number of data coders	Two (A.K. and K.K.)
25. Description of coding tree	For data analysis, two authors (A.K. and K.K.) repeatedly reviewed the interview transcript to identify inductively emergent codes and to assess connections amongst the codes to identify themes. During this process, they made a codebook with code labels and definitions. The codebook was refined iteratively with repeat transcript coding by and consultative discussions with the authors to ensure methodological rigor.
26. Derivation of themes	Themes were derived from the data. A.K., K.K., and the other authors discussed the emergence of the themes during the data analysis process.
27. Software	Excel
28. Participant checking	No feedback was sought from participants on the findings
Reporting	
29. Quotations presented	Yes, including the participant reference number
30. Data and findings consistent	Yes
31. Clarity of major themes	(1) Needs (2) Expectations (3) Barriers
32. Clarity of minor themes	(1) Needs Intrinsic factors Environmental factors (2) Expectations Medication review/reconciliation Education/counseling (3) Barriers Authorization Finance Professional Development Awareness
